# Multi-Morphology CeO_2_ Synthesis via Synergistic Induction by Solvent and Ammonium Bicarbonate

**DOI:** 10.3390/molecules31010116

**Published:** 2025-12-29

**Authors:** Yaohui Xu, Yu Hu, Sihan Li, Xiaoyu Gong, Shiya Xiao, Xin Zhang, Lian Li, Yanxi Liu, Zhao Ding

**Affiliations:** 1 Laboratory for Functional Materials, School of New Energy Materials and Chemistry, Leshan Normal University, Leshan 614000, China; xyh1986@lsnu.edu.cn (Y.X.); huyu82@lsnu.edu.cn (Y.H.); lisihan0769@163.com (S.L.); zxhy14@outlook.com (X.G.); xsy04021600@163.com (S.X.); zx524130@163.com (X.Z.); ll1010992025@163.com (L.L.); liuyanxi050127@163.com (Y.L.); 2 Leshan West Silicon Materials Photovoltaic and New Energy Industry Technology Research Institute, Leshan 614000, China; 3 National Engineering Research Center for Magnesium Alloys, College of Materials Science and Engineering, Chongqing University, Chongqing 400044, China

**Keywords:** CeO_2_, morphological control, solvothermal synthesis, ammonium bicarbonate, solvent effects, template-free synthesis

## Abstract

CeO_2_ is a crucial functional material in catalysis and energy applications, whose performance is highly morphology-dependent. Traditional synthesis methods often rely on organic templates or surfactants, which complicate the processes and pose environmental concerns. This study introduces an eco-friendly approach utilizing a methanol–water (MeOH-H_2_O) mixed solvent system combined with NH_4_HCO_3_ to achieve controllable synthesis of multi-morphology CeO_2_ without surfactants or templates. The effects of different solvent systems (pure H_2_O, pure MeOH, and their mixtures) and NH_4_HCO_3_ as an inexpensive regulator on precursor phase behavior and crystallization were systematically investigated. By optimizing the Ce:N molar ratios (1:1 to 1:7) as well as reaction times (0.5 to 36 h), our findings indicate that H_2_O significantly enhances crystallinity (from 40.9% to 61.4% for precursors, reaching 70.3% after calcination) and promotes octahedra formation in the MeOH-H_2_O mixed system, while NH_4_HCO_3_ acts as a structure-directing agent to control size (e.g., ~240 nm octahedra at Ce:N = 1:1, up to 375 nm at Ce:N = 1:2) and partially substitutes for high-temperature calcination in improving crystallinity. Variety morphologies, including plates, dendrites, octahedra, and hollow structures, were successfully synthesized. This work elucidates the synergistic mechanism by which solvents and NH_4_HCO_3_ influence CeO_2_ nucleation and growth, thereby providing an environmentally friendly synthesis route with significant potential applications in catalysis and energy storage.

## 1. Introduction

Cerium dioxide (CeO_2_), a versatile rare-earth metal oxide, demonstrates extensive application potential in various fields such as catalysis [1], energy storage [2] and environmental remediation [3]. In particular, its potential in advanced catalytic systems like single-atom catalysts has attracted growing attention [4]. This is attributed to its unique redox properties, exceptional oxygen storage capacity and structural flexibility [5]. The performance of CeO_2_ is largely influenced by its morphological characteristics, including specific surface area, exposed crystal facets, and particle size [6]. These factors directly affect its surface reactivity, mass transfer efficiency, and catalytic stability [7].

To date, numerous methods have been employed to synthesize CeO_2_ materials with diverse morphologies. Techniques such as microemulsion [8], hydrothermal treatment [9], sol–gel synthesis [10], Pechini process [11], and precipitation methods [12] have been utilized. However, these approaches often rely heavily on surfactants or templating agents, such as cetyltrimethylammonium bromide (CTAB) [13], amino acids [14], poly(methyl methacrylate) (PMMA) [15], powdered cellulose [16], polyvinyl alcohol (PVA)/polyvinylpyrrolidone (PVP) [17], and polystyrene spheres [18]. For instance, Ho [19] et al. utilized PVP to synthesize uniform CeO_2_ nanospheres, microrods, and spindle-like particles. Song [20] et al. prepared hollow CeO_2_ spheres via an oleic acid (OLA)-assisted solvothermal method. Kaviyarasu [21] et al. employed CTAB in ethanol to synthesize hexagonal cubic-shaped CeO_2_ nanofibers via a solvothermal method. Li [22] et al. fabricated three-dimensional hierarchical flower-like CeO_2_ microspheres using sodium dodecyl sulfonate and PEG 600 as structure-directing agents under mild conditions. Such heavy reliance on organic additives not only increases process complexity and production cost (due to the need for high-purity reagents and subsequent removal steps) but also introduces environmental concerns and post-treatment challenges. Furthermore, certain traditional surfactants, such as CTAB, demonstrate limited biodegradability and are susceptible to leaving residues during wastewater treatment processes. These residues may pose toxic effects on aquatic ecosystems [23,24]. Additionally, some ionic surfactants can decompose under high-temperature or extreme pH conditions, resulting in the formation of toxic by-products. For example, degradation intermediates of alkyl benzene sulfonate have been shown to carry genetic toxicity risks [25,26]. In recent years, there has been an increasing focus on developing green surfactants, such as bio-based or biodegradable alternatives [27,28], yet their efficiency and applicability in nanomaterial synthesis still lag behind those of traditional systems [29,30]. This underscores the urgent need for the development of new environmental control strategies.

Currently, the surfactant- and template-free synthesis of CeO_2_ has garnered increasing attention as a promising alternative strategy. For instance, Yu [31] et al. developed a template- and surfactant-free method to synthesize ultrathin CeO_2_ nanowires by refluxing at 140 °C for 12 h in a mixed ethanol–water solvent, utilizing 30% NH_3_·H_2_O as the precipitant. Chen [32] et al. successfully prepared single-crystalline-like CeO_2_ hollow nanocubes through a solvothermal approach at 160 °C for 9 h, employing peroxyacetic acid as an oxidizing agent without any templates. In our previous work, we established an oxidation-induced strategy for the template-free hydrothermal synthesis of mesoporous CeO_2_ [33,34,35,36]. It was observed that organic solvents such as ethylene glycol significantly influence the morphological evolution of CeO_2_ in template-free systems. Specifically, within a simple hydrothermal system comprising only water, ethylene glycol, and Ce(NO_3_)_3_·6H_2_O, the morphology can be tailored to solid spheres, hollow spheres, or multilayered structures by adjusting various synthesis parameters [37]. Nevertheless, the interplay between key synthesis parameters (such as precursor ratio, solvent composition and reaction time) and the dynamic phase evolution during template-free synthesis remains inadequately understood [38]. This knowledge gap considerably limits the rational design of CeO_2_ with specific morphologies.

Based on this, the present study proposes a novel synthesis strategy: by introducing environmentally benign and readily available ammonium bicarbonate (NH_4_HCO_3_) into a MeOH–H_2_O mixed solvent system, we achieve the controllable preparation of CeO_2_ with diverse morphologies without the use of any surfactants or templates. The synergistic effects of solvent composition, Ce/N molar ratio (1:1–1:7), and reaction time (0.5–36 h) on the crystal phase evolution and morphological control of CeO_2_ were systematically investigated. This work not only provides a new pathway for the environmentally friendly and efficient synthesis of CeO_2_ but also offers theoretical insights into crystal growth and morphology control under template-free conditions.

## 2. Results and Discussion

### 2.1. Influence of Solvothermal Reaction Media

The mineralization behavior of Ce(NO_3_)_3_·6H_2_O, as well as the phase composition and morphology of the resultant products, were first examined in pure H_2_O, MeOH, and a mixed solvent system of MeOH-H_2_O without the addition of NH_4_HCO_3_. Experimental results revealed no precipitate formation after solvothermal reactions in the pure aqueous medium, indicating that H_2_O exhibits limited efficacy in mineralizing Ce^3+^ ions and is therefore unsuitable as a reaction medium for this synthesis.

**Figure 1:**
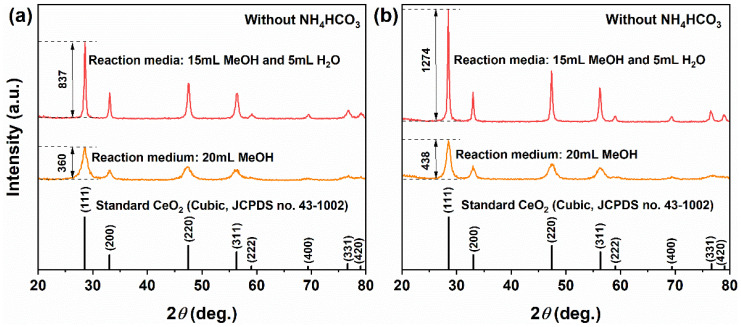
XRD patterns of samples synthesized solvothermally at 180 °C for 12 h (without NH_4_HCO_3_) in different solvent systems: (**a**) as-synthesized precursor and (**b**) after calcination in air at 500 °C for 1 h. Solvent systems: pure MeOH (20 mL) and MeOH–H_2_O mixed solvent (15 mL MeOH + 5 mL H_2_O).

Figure 1a presents the XRD patterns of precursors obtained through solvothermal processes in 20 mL pure MeOH and in a mixed solvent comprising 15 mL MeOH combined with 5 mL H_2_O. In the pure MeOH system, the precursor displayed four distinct diffraction peaks at 28.57°, 32.11°, 47.30° and 56.25°, which correspond to the (111), (200), (220) and (311) crystal planes of cubic fluorite-structured CeO_2_ (JCPDS No. 43-1002). The diffraction intensity observed for the (111) plane was measured at 360. Although other peaks were relatively weak, they remained identifiable and aligned closely with standard patterns for CeO_2_; thus confirming that the product consisted predominantly of single-phase CeO_2_, albeit featuring relatively low crystallinity. In contrast, when prepared within the MeOH–H_2_O mixed solvent system, the precursor exhibited eight clear diffraction peaks that fully matched those outlined in standard cubic CeO_2_ patterns corresponding to crystal planes (111), (200), (220), (311), (222), (400), (331), and (420). No impurity phases were detected under these conditions. Notably, there was an increase in diffraction intensity for the (111) plane up to 837, a significant enhancement of approximately 132.5%, which suggests improved crystallinity attributed to the facilitating role of H_2_O in promoting Ostwald ripening processes along with better crystal development.

After calcination at 500 °C for 1 h (Figure 1b), all samples retained the characteristic diffusion patterns of pure cubic CeO_2_; however, both the intensity and sharpness of the peaks increased significantly. For the CeO_2_ derived from the pure MeOH system, the diffraction intensity of the (111) plane rose from 360 to 438, representing an increase of 21.7%. In contrast, for the sample obtained from a MeOH–H_2_O mixed solvent, it escalated from 837 to 1274, marking a substantial increase of 52.2%. These results indicate that incorporating H_2_O not only facilitates the formation of highly crystalline CeO_2_ during the solvothermal process but also enhances its crystallinity further through subsequent calcination. This finding is crucial for achieving the controlled synthesis of CeO_2_ crystallinity and has important implications for its applications in catalysis and other fields. The crystallinity and exposed facets are known to significantly affect the electronic structure and surface reactivity of metal oxides, as demonstrated in doped nickel oxide systems for electrocatalysis [39].

**Figure 2:**
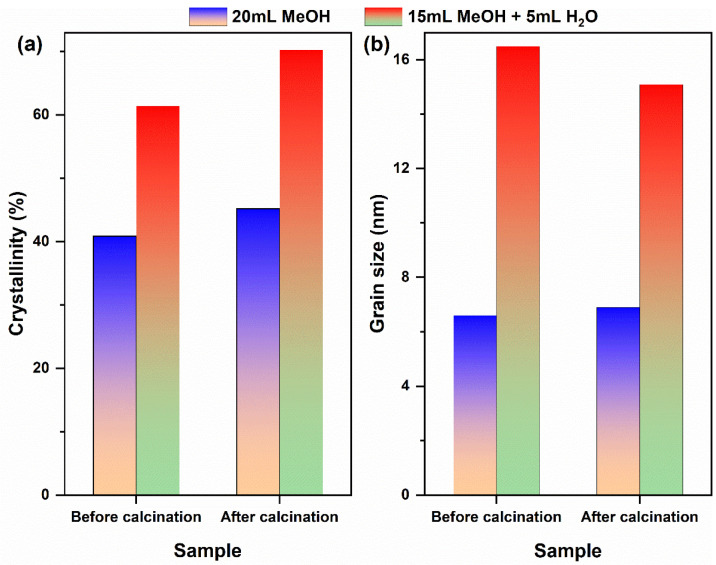
(**a**) Crystallinity and (**b**) grain size of CeO_2_ samples synthesized solvothermally at 180 °C for 12 h (without NH_4_HCO_3_) in different reaction media, before and after calcination in air at 500 °C for 1 h. Solvent systems: pure MeOH (20 mL) and MeOH–H_2_O mixed solvent (15 mL MeOH + 5 mL H_2_O).

Although single-phase cubic CeO_2_ can be synthesized via a one-step solvothermal reaction (180 °C, 12 h) in both the pure MeOH and MeOH–H_2_O mixed solvent systems, the phase remains unchanged after calcination. However, the composition of the solvent significantly influences the mineralization and crystallization behavior of Ce^3+^, as well as the crystal quality of the final product. Therefore, the crystallinity and grain size of both the solvothermally derived precursors and the calcined CeO_2_ were further evaluated, as depicted in Figure 2. In the pure MeOH system (Figure 2a), the crystallinity of both the precursor and calcined CeO_2_ was measured at 40.9% and 45.2%, respectively, with corresponding grain sizes of 6.6 nm and 6.9 nm. This indicates that thermodynamically stable nanocrystals were formed during the solvothermal process; moreover, calcination did not induce significant grain growth. In contrast, within the MeOH–H_2_O mixed solvent system (Figure 2b), the crystallinity of the precursor registered at 61.4%, which increased to 70.3% after calcination; concurrently, there was a slight decrease in grain size from 16.5 nm to 15.1 nm. This reduction may be attributed to either the removal of amorphous components or repair of structural defects during calcination, both contributing to further enhancement in crystal integrity. A comprehensive summary of these structural parameters, including the calculated strain derived from XRD peak broadening analysis, is provided in Table 1. The notably lower strain value for the CeO_2_ sample from the MeOH-H_2_O system quantitatively supports the role of H_2_O in promoting the growth of more relaxed and defect-annealed crystals.

**Figure 3:**
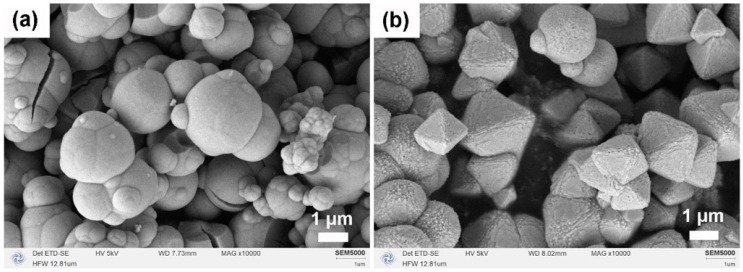
SEM images of CeO_2_ samples synthesized solvothermally at 180 °C for 12 h (without NH_4_HCO_3_) in different solvent systems after calcination in air at 500 °C for 1 h: (**a**) pure MeOH (20 mL) and (**b**) MeOH–H_2_O mixed solvent (15 mL MeOH + 5 mL H_2_O).

Figure 3 illustrates the micro-morphologies of CeO_2_ produced after calcination in two different solvent systems. In the pure MeOH system (Figure 3a), CeO_2_ particles exhibited spherical hard aggregates due to incomplete Ostwald ripening. Conversely, in the MeOH–H_2_O mixed solvent system (Figure 3b), the addition of H_2_O effectively inhibited hard aggregation, enhanced particle monodispersity, and facilitated the formation of regular micron-sized octahedral structures with edge lengths less than 3 μm. This observation suggests that H_2_O plays a crucial role in mediating chemical reactions between Ce^3+^ ions and MeOH, guiding the transformation of agglomerates into a thermodynamically more stable octahedral morphology.

### 2.2. Influence of a Small Amount of NH_4_HCO_3_

**Figure 4:**
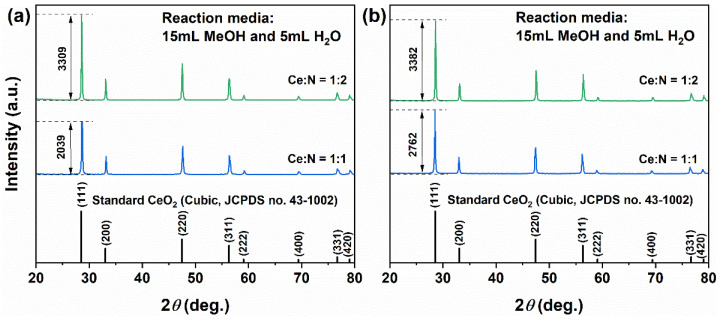
XRD patterns of samples synthesized solvothermally at 180 °C for 12 h in a mixed solvent of 15 mL MeOH and 5 mL H_2_O with Ce:N molar ratios of 1:1 and 1:2, (**a**) before and (**b**) after calcination in air at 500 °C for 1 h.

Based on the aforementioned results, a mixed solvent system comprising 15 mL MeOH and 5 mL H_2_O was selected to further examine the impact of adding a small quantity of NH_4_HCO_3_ on the products. The molar ratios of Ce:N were established at 1:1 and 1:2. As illustrated in Figure 4a, following the incorporation of NH_4_HCO_3_, the solvothermally obtained precursors retained a phase-pure cubic CeO_2_ structure (JCPDS No. 43-1002), indicating that the introduction of NH_4_HCO_3_ did not alter the crystal structure. However, a significant increase in diffraction peak intensities was observed (reaching values of 2039 and 3309, respectively), suggesting notably enhanced crystallinity, likely attributable to optimized crystal growth within an alkaline environment.

After calcination at 500 °C (Figure 4b), the phase composition of all samples remained unchanged. When the molar ratio of Ce:N was set at 1:1, the diffraction intensity increased to 2762, indicating an enhancement in crystallinity. In contrast, at a Ce:N molar ratio of 1:2, the intensity remained essentially stable (3382), suggesting that an appropriate amount of NH_4_HCO_3_ can partially substitute for the crystallinity-enhancing effect provided by high-temperature calcination.

**Figure 5:**
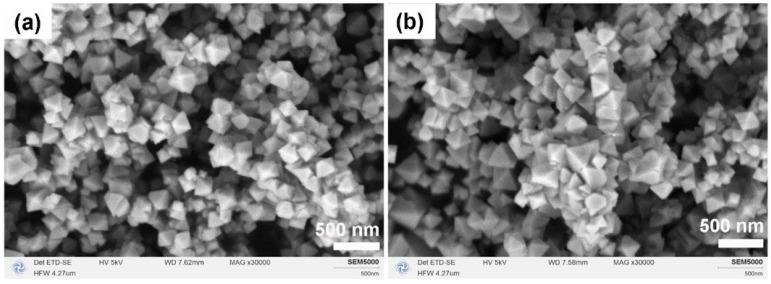
SEM images of CeO_2_ samples synthesized solvothermally at 180 °C for 12 h in a mixed solvent of 15 mL MeOH and 5 mL H_2_O with Ce:N molar ratios of (**a**) 1:1 and (**b**) 1:2, after calcination in air at 500 °C for 1 h.

Following the introduction of NH_4_HCO_3_, the CeO_2_ products preserved their octahedral morphology but underwent a significant reduction in size from the micron-scale to the nano-scale, as revealed by SEM analysis (Figure 5). Specifically, at a Ce:N molar ratio of 1:1, the resulting octahedra were relatively uniform, with an average edge length of approximately 240 nm (Figure 5a. Conversely, at a Ce:N molar ratio of 1:2, the particle size distribution exhibited slight broadening, with maximum edge lengths reaching up to 375 nm (Figure 5b). These findings suggest that NH_4_HCO_3_ functions as an effective structure-directing agent, and that its dosage can further modulate both the particle size and the uniformity of the resulting CeO_2_ nanocrystals.

### 2.3. Influence of Ce:N Molar Ratios

**Figure 6:**
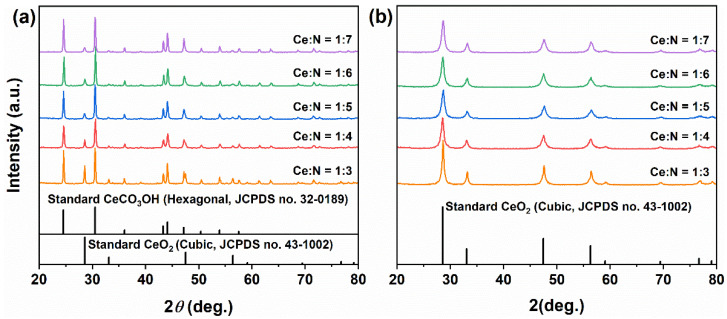
XRD patterns of samples synthesized solvothermally at 180 °C for 12 h with different Ce:N molar ratios of 1:3, 1:4, 1:5, 1:6, and 1:7 (**a**) before and (**b**) after calcination in air at 500 °C for 1 h.

To further investigate the impact of NH_4_HCO_3_ dosage on the phase composition and morphology of the products, we increased the Ce:N molar ratio to 1:3–1:7 within a fixed mixed solvent system (15 mL MeOH + 5 mL H_2_O). Figure 6a presents the XRD patterns of the solvothermal precursors at varying ratios. At a Ce:N ratio of 1:3, alongside cubic CeO_2_ (JCPDS No. 43-1002), diffraction peaks corresponding to hexagonal CeCO_3_OH (JCPDS No. 32-0189) emerged and were predominant. As the Ce:N ratio was raised to 1:5, there was an intensified presence of CeCO_3_OH diffraction peaks accompanied by a reduction in intensity for those associated with CeO_2_. This trend indicates that NH_4_HCO_3_ facilitates both the dissolution of CeO_2_ and promotes the formation of the CeCO_3_OH phase. When further increasing the Ce:N ratio to 1:7, stabilizing diffraction intensities for both phases suggested that an equilibrium between dissolution and recrystallization had been attained. Following calcination (Figure 6b), all samples completely converted into pure cubic CeO_2_, thus confirming that the intermediate phase of CeCO_3_OH can be entirely decomposed into CeO_2_ via thermal treatment.

**Figure 7:**
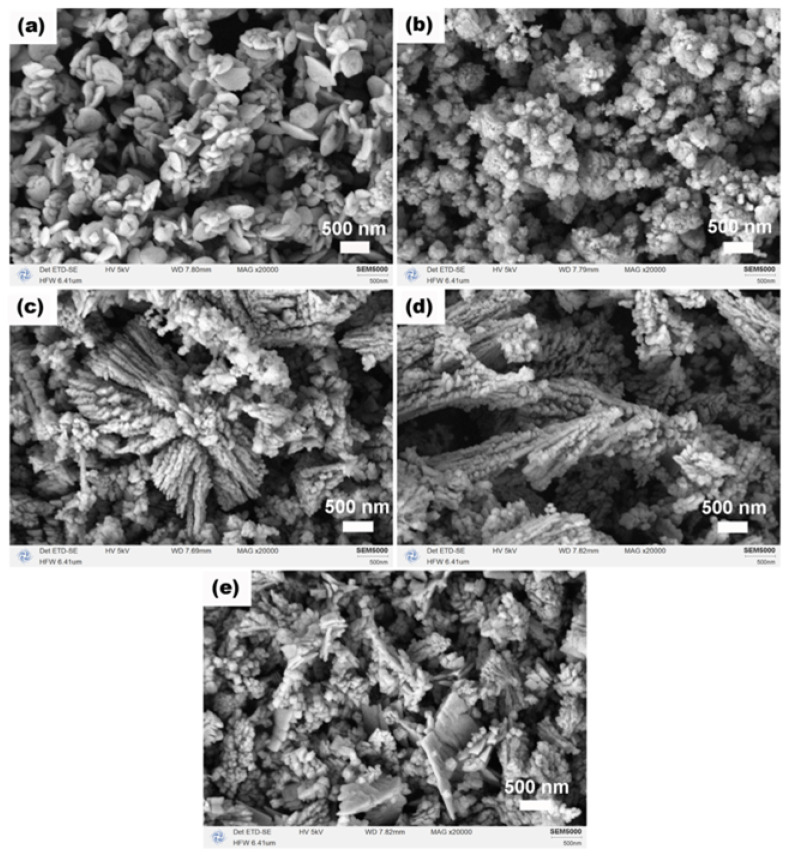
SEM images of CeO_2_ samples synthesized solvothermally at 180 °C for 12 h with different Ce:N molar ratios of (**a**) 1:3, (**b**) 1:4, (**c**) 1:5, (**d**) 1:6, and (**e**) 1:7 after calcination in air at 500 °C for 1 h.

The morphologies of CeO_2_ obtained after calcination at various Ce:N molar ratios are illustrated in Figure 7. At a Ce:N ratio of 1:3 (Figure 7a), the product predominantly comprised nanodisks with diameters approximately 500 nm (with an aspect ratio, thickness/diameter, estimated from representative areas to be about 0.1–0.2), accompanied by a limited number of nano-octahedra (edge length < 350 nm). Increasing the ratio to 1:4 (Figure 7b) resulted in a transformation into aggregates of nanoparticles displaying discernible porous structures, likely attributed to Ostwald ripening or dissolution–recrystallization processes. The primary nanoparticles within these aggregates ranged from ~20 to 80 nm in size. At a Ce:N ratio of 1:5 (Figure 7c), dendritic structures formed from nanoparticles emerged alongside some aggregated particles; this may be due to the excessive presence of NH_4_HCO_3_, which potentially heightened the supersaturation level of the solution and facilitated oriented attachment among nanoparticles. The dendritic branches are typically 50–80 nm in width and several micrometers in length. Similar morphological evolution from plates to dendrites driven by additive concentration has been observed in other metal oxide systems [40]. As we progressed to a Ce:N ratio of 1:6 (Figure 7d), the aggregates were no longer present, while dendritic structures continued to develop further, indicating that NH_4_HCO_3_ promotes the establishment of a new dissolution–recrystallization equilibrium within the precursor phase. However, when increasing the Ce:N ratio to 1:7 (Figure 7e), there was an observed reduction in size for dendritic structures along with the re-emergence of irregular plate-like forms and aggregates composed of nano-octahedra, suggesting that an excess amount of NH_4_HCO_3_ might disrupt the original crystallization kinetics by introducing multiple competing growth pathways during morphological evolution of the precursor phase. To correlate these morphological changes with structural characteristics, key parameters derived from XRD for all Ce:N ratios are compiled in Table 2. Of particular note is the trend in lattice strain, which increased significantly with higher NH_4_HCO_3_ content, providing a structural rationale for the observed transition from well-defined octahedra to complex dendritic and aggregated morphologies.

### 2.4. Influence of Solvothermal Reaction Time

**Figure 8:**
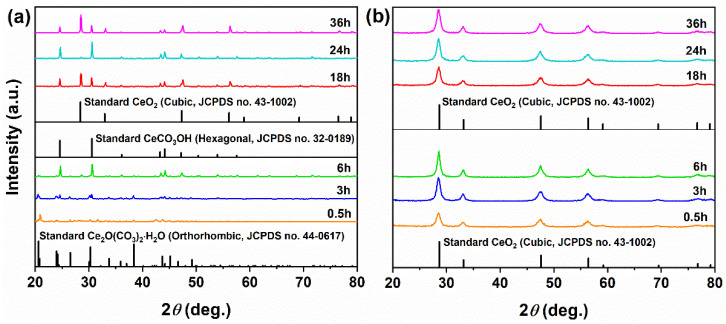
XRD patterns of samples synthesized solvothermally at 180 °C for different reaction times of 0.5, 3, 6, 18, 24, and 36 h with a Ce:N molar ratio of 1:3 (**a**) before and (**b**) after calcination in air at 500 °C for 1 h.

The influence of reaction time (0.5–36 h) on phase evolution and morphology was systematically investigated in the mixed solvent system (15 mL MeOH + 5 mL H_2_O) at a Ce:N ratio of 1:3. After the shortest reaction time of 0.5 h (Figure 8a), the main phase of the precursor obtained through solvothermal synthesis was orthorhombic Ce_2_O(CO_3_)_2_·H_2_O (JCPDS No. 44-0617). When the reaction time was extended to 3 h, Ce_2_O(CO_3_)_2_·H_2_O remained the dominant phase, but the hexagonal CeCO_3_OH phase (JCPDS No. 32-0189) also appeared. At 6 h, the diffraction peaks of Ce_2_O(CO_3_)_2_·H_2_O became very weak, while CeCO_3_OH became the major phase, accompanied by the emergence of cubic CeO_2_. After 18 h, the Ce_2_O(CO_3_)_2_·H_2_O phase disappeared, and CeO_2_ became dominant, with CeCO_3_OH as the secondary phase. Interestingly, at 24 h, the diffraction peaks of CeCO_3_OH intensified and became dominant again, possibly due to transient reaction conditions such as local pH fluctuations or incomplete consumption of precursors. After 36 h, the system returned to being dominated by CeO_2_, with CeCO_3_OH as the minor phase, indicating that prolonged reaction time drives the system toward thermodynamic equilibrium, as CeO_2_ was the most stable phase under oxidative conditions. After calcination at 500 °C (Figure 8b), all precursors were completely converted to cubic CeO_2_, consistent with their thermal decomposition pathways, with no residual precursor phases observed. Furthermore, variations in XRD peak width and intensity among the CeO_2_ samples may originate from differences in inherited grain size or defect density from the precursor compositions.

**Figure 9:**
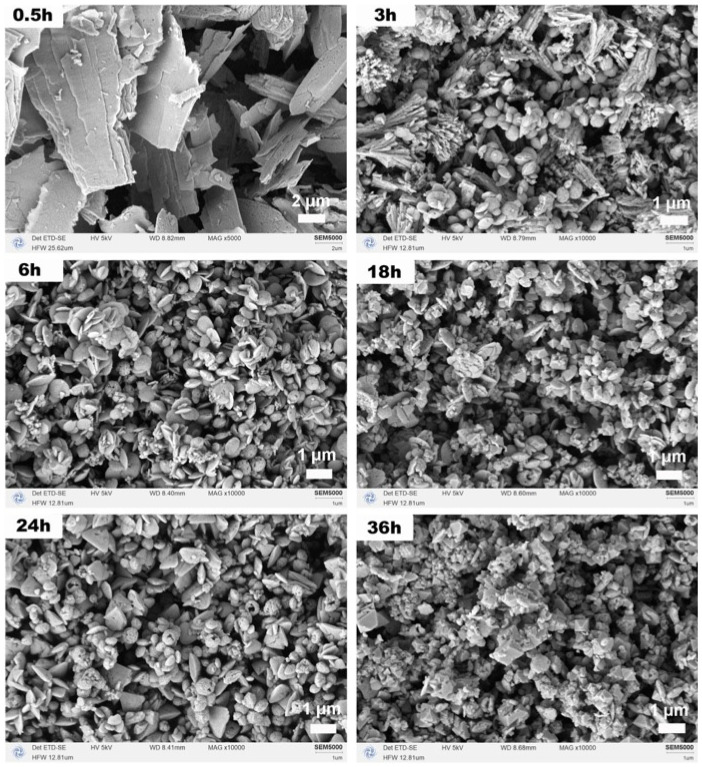
SEM images of CeO_2_ samples synthesized solvothermally at 180 °C for different reaction times of 0.5, 3, 6, 18, 24, and 36 h with a Ce:N molar ratio of 1:3 after calcination in air at 500 °C for 1 h.

Figure 9 illustrates the morphological evolution of CeO_2_ samples following calcination at various solvothermal reaction times. After 0.5 h, the predominant morphology consisted of micron-sized plate-like structures formed from nanoscale sheets, likely resulting from rapid nucleation under conditions of high supersaturation. These primary sheets have a thickness on the order of tens of nanometers and lateral dimensions of 1–10 μm. At 3 h, a coexistence of plate-like and dendritic structures was observed. By 6 h, the morphology transitioned to nanodisk structures. These nanodisks measured ~300–600 nm in diameter, consistent with the dimensions observed in the Ce:N series (Figure 7a). After 18 h, nano-octahedra emerged alongside disk-like structures. Notably, at 24 h, triangular plate-like (with side lengths ~1 μm) and hollow spherical structures (with diameters ~500 nm) appeared while the quantity of disk-like structures diminished; this observation indicates transient dissolution–recrystallization and Ostwald ripening processes were occurring. Following a duration of 36 h, the morphology predominantly reverted to octahedral forms, which is consistent with the XRD results and confirms the thermodynamic stability of CeO_2_ under prolonged reaction conditions. The octahedra at this stage are well-defined with edge lengths ranging from 100 to 500 nm. The quantitative evolution of crystallographic parameters with reaction time is detailed in Table 3. The data revealed a systematic decrease in lattice strain over time, which aligns with the progression from kinetically formed metastable phases (e.g., carbonate intermediates) towards the thermodynamically stable, low-strain CeO_2_ octahedra.

In summary, the evolution of the final CeO_2_ morphology, from plates, dendrites, and hollow structures to octahedra, triangular plates, and hollow architectures, underscores the intricate interplay between dynamic and thermodynamic factors during nanomaterial growth. The ultimate morphology is significantly influenced by both the chemical composition of the precursor and the chemical reactions that take place throughout the solvothermal process. Consequently, potential chemical reactions and processes that may occur during this solvothermal reaction have been proposed and are summarized in Equations (1)–(15).

First, the potential interaction between Ce^3+^ ions and MeOH, as illustrated in Equations (1)–(3), facilitates the crystallization of CeO_2_ under the influence of O_2_. This oxygen originates from air trapped within the reactor and from oxygen dissolved in the solvent. This observation elucidates why CeO_2_ can be synthesized in MeOH or MeOH–H_2_O systems without requiring the addition of NH_4_HCO_3_. It is noted that MeOH can be oxidized on CeO_2_ surfaces at elevated temperatures (e.g., ~200 °C) [41], which may influence the local redox environment during synthesis. Furthermore, the H_2_O and NO_3_^−^ from Ce(NO_3_)_3_·6H_2_O are not merely spectators; they can participate in the reaction network. H_2_O can mediate the ion solvation and hydrolysis, while NO_3_^−^ can act as an inherent oxidant during thermal decomposition, potentially generating species such as NO_2_, N_2_O_5_, and O_2_ at the applied temperatures, thereby contributing to the oxidation of Ce^3+^.

(1) $${\mathrm{Ce}}^{3+}{+3(\mathrm{HOCH}}_{3})\;⇄{\mathrm{Ce}(\mathrm{OCH}}_{3}{)}_{3}+3H^{+}$$

(2) $${\mathrm{Ce}(\mathrm{OCH}}_{3}{)}_{3}{+3(\mathrm{HOCH}}_{3})\;⇄{\;\mathrm{Ce}(\mathrm{OH})}_{3}{+3(\mathrm{CH}}_{3}{\mathrm{OCH}}_{3})$$

(3) $${\;\mathrm{Ce}(\mathrm{OH})}_{3}\;\overset{O_{2}}{⇄}{\;\mathrm{CeO}}_{2}$$

Subsequently, the initially formed CeO_2_ nanocrystals may serve as catalysts for reactions involving MeOH molecules, potentially resulting in chemical transformations as described in Equations (4)–(7). These processes generate derivatives such as HCHO and HCOOH. The resultant CeO_2_ may subsequently dissolve under the influence of HCOOH, as indicated in Equations (8) and (9). Furthermore, H_2_O serves a mediating role by participating in chemical interactions between Ce^3+^ ions and MeOH, possibly via Equation (8). This process likely facilitates the transformation of hard agglomerates into octahedral structures, which is supported by the findings presented in Figure 1, Figure 2 and Figure 3.

(4) $${2(\mathrm{HOCH}}_{3})\;\overset{{\mathrm{CeO}}_{2}}{⇄}{\;\mathrm{CH}}_{3}{\mathrm{OCH}}_{3}+H_{2}O$$

(5) $${2(\mathrm{HOCH}}_{3})+O_{2}\;\overset{{\mathrm{CeO}}_{2}}{⇄}\;2CO_{2}↑+4H_{2}O$$

(6) $${2(\mathrm{HOCH}}_{3})+O_{2}\;\overset{{\mathrm{CeO}}_{2}}{⇄}\;2HCHO+2H_{2}O$$

(7) $${\mathrm{HOCH}}_{3}+O_{2}\;\overset{{\mathrm{CeO}}_{2}}{⇄}\;\mathrm{HCOOH}+H_{2}O$$

(8) $$\mathrm{HCOOH}\overset{\mathrm{Ionization}}{\underset{H_{2}O}{⇄}}{\mathrm{COOH}}^{-}+H^{+}$$

(9) $${4\mathrm{CeO}}_{2}+12H^{+}⇄\;{4\mathrm{Ce}}^{3+}+6H_{2}O+O_{2}$$

After the addition of NH_4_HCO_3_ to the MeOH–H_2_O mixed solvent system, three processes involving NH_4_HCO_3_ occur: thermal decomposition (Equation (10)), ionization (Equations (10) and (11)), and hydrolysis (Equations (12) and (13)). On the one hand, these processes lead to the generation of gases such as NH_3_ and CO_2_, which further increases the pressure within the solvothermal reaction system. This elevation in pressure facilitates product precipitation or dissolution–recrystallization processes along with Ostwald ripening mechanisms, while significantly enhancing the crystallinity of precipitated crystals. A comparison of the XRD shown in Figure 4a,b supports this hypothesis: under Ce:N molar ratios of 1:1 and 1:2, CeO_2_ produced via the solvothermal process exhibited no significant changes before and after calcination, indicating that introducing an appropriate amount of NH_4_HCO_3_ can substitute for high-energy-consuming calcination process. On the other hand, these processes also yield species including CO_3_^2−^, OH^−^, H^+^, H_2_CO_3_, and NH_4_OH, thus creating favorable conditions for crystallizing initial grains such as Ce_2_O(CO_3_)_2_·H_2_O (Equation (14)) alongside the subsequent formation of CeCO_3_OH intermediates (Equation (15)). Notably, it is important to recognize that the thermodynamic processes described in Equations (3), (14) and (15) exist in a delicate yet complex dynamic equilibrium. Herein dissolution and recrystallization occur simultaneously while influenced by various factors such as pH level of the system, concentration of CO_3_^2−^ ions, pressure conditions, and reaction duration, potentially leading to coexistence among three phases at certain stages, as depicted in Figure 8a at 180 °C for 6 h. Among the proposed pathways, the oxidation of Ce^3+^ (Equations (1)–(3)), the decomposition and hydrolysis of NH_4_HCO_3_ (Equations (10)–(13)), and the formation/decomposition of the intermediate carbonate phases (Equations (14)–(15)) are considered the key steps governing phase evolution and morphology control.

(10) $${\mathrm{NH}}_{3}↑+CO_{2}↑+H_{2}O\overset{\mathrm{Thermolysis}}{⇄}{\;\mathrm{NH}}_{4}{\mathrm{HCO}}_{3}\overset{\mathrm{Ionization}}{⇄}{\mathrm{NH}}_{4}^{+}+HCO_{3}^{-}$$

(11) $${\mathrm{HCO}}_{3}^{-}\overset{\mathrm{Ionization}}{⇄}\;{\mathrm{CO}}_{3}^{2-}+H^{+}$$

(12) $${\mathrm{HCO}}_{3}^{-}+H_{2}O\overset{\mathrm{Hydrolysis}}{⇄}H_{2}{\mathrm{CO}}_{3}+{\mathrm{OH}}^{-}\;(H_{2}{\mathrm{CO}}_{3}⇄{\mathrm{CO}}_{2}↑+H_{2}O)$$

(13) $${\mathrm{NH}}_{4}^{+}+H_{2}O\overset{\mathrm{Hydrolysis}}{⇄}{\mathrm{NH}}_{4}\mathrm{OH}+H^{+}\;({\mathrm{NH}}_{4}\mathrm{OH}⇄{\mathrm{NH}}_{3}↑+H_{2}O)$$

(14) $${2\mathrm{Ce}}^{3+}+2{\mathrm{OH}}^{-}+2{\mathrm{CO}}_{3}^{2-}⇄\;{\mathrm{Ce}}_{2}{O(\mathrm{CO}}_{3}{)}_{2}\cdot H_{2}O↓$$

(15) $${\mathrm{Ce}}^{3+}+{\mathrm{CO}}_{3}^{2-}+{\mathrm{OH}}^{-}⇄\;{\mathrm{CeCO}}_{3}\mathrm{OH}↓$$

## 3. Materials and Methods

### 3.1. Materials

Cerium nitrate hexahydrate (Ce(NO_3_)_3_·6H_2_O, 99.95%) and ammonium bicarbonate (NH_4_HCO_3_, 99.995%) were purchased from Aladdin Co. Ltd. (Shanghai, China). Methanol (MeOH, analytical grade, 99.5%) was supplied by Chengdu Kelong Chemical Co. Ltd. (Chengdu, China). Deionized water (H_2_O) was obtained from an HRO-402 ultrapure water system (Chengdu Zhonghan Water Treatment Equipment Co., Ltd., Chengdu, China) and used throughout all experiments.

### 3.2. Synthesis of CeO_*2*_ Powders

**Figure 10:**
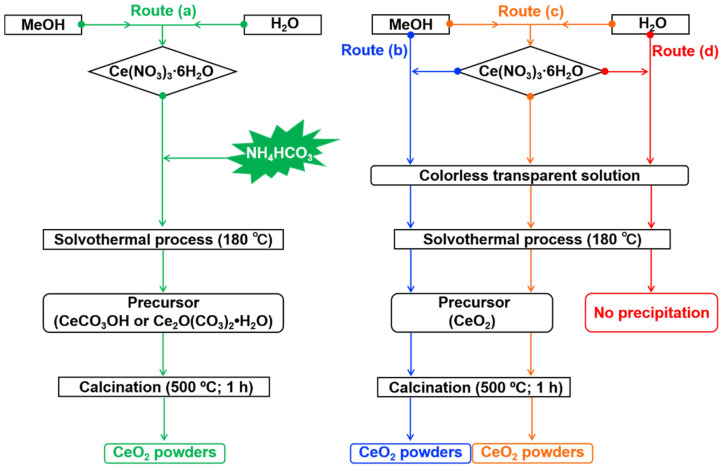
Schematic illustration of the synthesis procedure and control experiments: (a) the primary route with NH_4_HCO_3_ in a mixed solvent of 15 mL MeOH and 5 mL H_2_O; control experiments (b), (c), and (d) conducted without NH_4_HCO_3_ in (b) 20 mL pure MeOH, (c) 15 mL MeOH + 5 mL H_2_O, and (d) 20 mL pure H_2_O (yielded no precipitate).

The synthesis procedure is illustrated in Figure 10. In a typical process (Route a), 4 mmol of Ce(NO_3_)_3_·6H_2_O and varying amounts of NH_4_HCO_3_ (corresponding to Ce:N molar ratios from 1:1 to 1:7) were added to a mixed solvent of 15 mL MeOH and 5 mL H_2_O. After stirring for 15 min, the solution was transferred into a Teflon-lined autoclave and reacted at 180 °C for different durations (0.5–36 h). The resulting precipitate (namely, “Precursor” in Figure 10) was collected, washed with deionized water until neutral pH, dried at 60 °C for 24 h, and finally calcined at 500 °C for 1 h in air (with a ramp rate of 10 °C/min) to obtain light-yellow CeO_2_ powders.

For comparison, three control experiments were performed without NH_4_HCO_3_: in 20 mL MeOH (Route b), 15 mL MeOH + 5 mL H_2_O (Route c), and 20 mL H_2_O (Route d). In contrast, no precipitate formed in the pure aqueous system (Route d). It should be noted that in the pure aqueous system (Route d), no precipitate was obtained under the given conditions, confirming the necessity of MeOH for precursor mineralization in this system.

### 3.3. Characterization

The crystalline phases of the precursors and final products were analyzed by X-ray diffraction (XRD, DX-2700, Dandong Haoyuan Instrument Co., Ltd., Dandong, China) using Cu Kα radiation. The morphologies of the samples were observed by scanning electron microscopy (SEM, SEM5000, CIQTEK Co., Ltd., Hefei, China).

## 4. Conclusions

CeO_2_ with diverse morphologies was controllably synthesized by adjusting the MeOH-H_2_O solvent composition, introducing NH_4_HCO_3_ (Ce:N = 1:1–1:7), and varying solvothermal reaction times (0.5–36 h). A single-phase cubic CeO_2_ can be produced via a one-step solvothermal reaction at 180 °C for 12 h in both pure MeOH and MeOH–H_2_O mixed systems; however, no precipitate forms in purely aqueous solutions, highlighting the critical role of solvent choice. The addition of H_2_O to MeOH significantly improved CeO_2_ crystallinity from 40.9% to 70.3% after calcination and transformed the morphology from spherical agglomerates into well-dispersed micron-sized octahedra (<3 μm). NH_4_HCO_3_ acted as both a structure-directing agent and a crystallization promoter: even at low concentrations (Ce:N = 1:1–1:2), it enabled precise size control, yielding nano-octahedra (~240 nm) and demonstrating potential to partly substitute high-temperature calcination for crystallinity optimization. At higher Ce:N ratios, morphology evolved from nanoplates (~500 nm at 1:3) to dendritic structures (1:4–1:6), revealing the role of NH_4_HCO_3_ in directing phase transformation via intermediate CeCO_3_OH. With extended reaction time, the CeO_2_ morphology progressed from plates and dendrites to hollow architectures and finally to thermodynamically stable octahedra, reflecting a complex interplay of kinetic and thermodynamic factors. This work clarifies the synergistic mechanism between solvent-mediated kinetics and NH_4_HCO_3_ pyrolysis chemistry in regulating CeO_2_ morphology, providing an eco-friendly, template-free strategy for designing morphology-tunable CeO_2_ materials with potential applications in catalysis and energy storage. To fully realize their potential in catalysis and energy storage, future efforts will focus on correlating the specific morphologies with functional properties and extending this synergistic strategy to other metal oxide systems.

## Figures and Tables

**Table 1:** Crystallite size and structural parameters of CeO_2_ synthesized in different solvent systems.

Sample Description	Condition	Crystallite Size (nm)	Relative Crystallinity (%)	Strain (%)
Precursor (Pure MeOH)	180 °C, 12 h, no NH_4_HCO_3_	6.6	40.9	1.678
CeO_2_ (Pure MeOH)	Calcined at 500 °C for 1 h	6.9	45.2	1.639
Precursor (MeOH–H_2_O)	180 °C, 12 h, no NH_4_HCO_3_	16.5	61.4	0.337
CeO_2_ (MeOH–H_2_O)	Calcined at 500 °C for 1 h	15.1	70.3	0.550

Note: Crystallite size (nm) was calculated from the (111) diffraction peak using the Scherrer equation.

**Table 2:** Crystallite size and structural parameters of CeO_2_ synthesized with different Ce:N molar ratios.

Ce:N Molar Ratio	Crystallite Size (nm)	Relative Crystallinity (%)	Strain (%)	Dominant Phase (Before Calcination)	Dominant Morphology (After Calcination)
1:1	15.5	79.6	0.152	CeCO_3_OH + CeO_2_	Nano-octahedra
1:2	17.2	88.2	0.121		Nano-octahedra
1:3	18.7	69.4	0.614		Nanodisks
1:4	9.7	67.9	1.084		Nanoparticle aggregates
1:5	10.5	61.9	1.110		Dendritic structures
1:6	11.1	66.9	0.854		Dendritic structures
1:7	12.2	68.7	0.830		Irregular plates/aggregates

**Table 3:** Crystallite size and structural parameters of CeO_2_ synthesized for different reaction times (Ce:N = 1:3).

Reaction Time (h)	Crystallite Size (nm)	Relative Crystallinity (%)	Strain (%)	Dominant Phase (Before Calcination)	Dominant Morphology (After Calcination)
0.5	8.3	23.2	0.956	Ce_2_O(CO_3_)_2_·H_2_O	Plate-like structures
3	9.6	60.1	0.804	Ce_2_O(CO_3_)_2_·H_2_O + CeCO_3_OH	Plates + Dendrites
6	10.6	68.3	0.605	CeCO_3_OH + CeO_2_	Nanodisks
18	9.8	62.1	0.722	CeO_2_ + CeCO_3_OH	Nano-octahedra + Disks
24	9.2	65.6	0.725	CeCO_3_OH	Triangular plates + Hollow spheres
36	9.1	69.5	0.762	CeO_2_ + CeCO_3_OH	Irregular octahedra

## Data Availability

Data are contained within the article.
